# YAP Activation in Promoting Negative Durotaxis and Acral Melanoma Progression

**DOI:** 10.3390/cells11223543

**Published:** 2022-11-09

**Authors:** Yuxing Huang, Jing Su, Jiayong Liu, Xin Yi, Fang Zhou, Jiaran Zhang, Jiaxiang Wang, Xuan Meng, Lu Si, Congying Wu

**Affiliations:** 1 Institute of Systems Biomedicine, Beijing Key Laboratory of Tumor Systems Biology, School of Basic Medical Sciences, Peking University Health Science Center, Peking University, Beijing 100191, China; 2 Center for Precision Medicine Multi-Omics Research, Peking University Health Science Center, Peking University, Beijing 100191, China; 3 Department of Pathology, School of Basic Medical Sciences, Third Hospital, Peking University Health Science Center, Beijing 100191, China; 4 Key Laboratory of Carcinogenesis and Translational Research (Ministry of Education/Beijing), Department of Bone and Soft Tissue Tumor, Peking University Cancer Hospital & Institute, Beijing 100142, China; 5 Key Laboratory of Carcinogenesis and Translational Research (Ministry of Education/Beijing), Department of Renal Cancer and Melanoma, Peking University Cancer Hospital & Institute, Beijing 100142, China; 6 Hepatobiliary Surgery Department, National Cancer Center, National Clinical Research Center for Cancer, Cancer Hospital, Chinese Academy of Medical Sciences and Peking Union Medical College, Beijing 100730, China; 7 Hebei Cancer Hospital, Chinese Academy of Medical Sciences, Langfang 065000, China; 8 Jiangsu Center for the Collaboration and Innovation of Cancer Biotherapy, Cancer Institute, Xuzhou Medical College, Xuzhou 221004, China

**Keywords:** negative durotaxis, YAP, RhoA-Myosin II, acral melanoma

## Abstract

Directed cell migration towards a softer environment is called negative durotaxis. The mechanism and pathological relevance of negative durotaxis in tumor progression still requires in-depth investigation. Here, we report that YAP promotes the negative durotaxis of melanoma. We uncovered that the RhoA-myosin II pathway may underlie the YAP enhanced negative durotaxis of melanoma cells. Acral melanoma is the most common subtype of melanoma in non-Caucasians and tends to develop in a stress-bearing area. We report that acral melanoma patients exhibit YAP amplification and increased YAP activity. We detected a decreasing stiffness gradient from the tumor to the surrounding area in the acral melanoma microenvironment. We further identified that this stiffness gradient could facilitate the negative durotaxis of melanoma cells. Our study advanced the understanding of mechanical force and YAP in acral melanoma and we proposed negative durotaxis as a new mechanism for melanoma dissemination.

## 1. Introduction

Matrix stiffening promotes the reorganization of actin cytoskeleton, enhancing glycolysis and tumor cell growth [1], which in turn affects extracellular matrix (ECM) crosslinking [2]. Meanwhile, external forces generated by matrix stiffening remodels chromatin structure and regulates gene expression [3]. Stiffer stroma induces the expression of oncogene ZNF217 to increase breast cancer risks [4], while stiffer ECM stiffness promotes IDH1-dependent HIF1α-tenascin C expression to regulate brain cancer [5]. Accumulative evidence also suggests that mechanical stress may play an important role in tumor metastasis [6,7]. Stiffened ECM has been reported to trigger the epithelial-mesenchymal transition and induce neural crest cell migration [8], while stiff substrate promotes pancreatic cancer spreading [9].

The ability of cells to sense and migrate along the gradients of substrate stiffness has been coined as durotaxis [10]. Contractile mechanosensation, the probing of the local substrate by actin-based protrusions, and FA signaling, are reported to be the mechanisms underlying durotaxis [11,12]. It has been reported recently that some cells managed to exert negative durotaxis or adurotactic migration [13,14]. A motor-clutch model has been proposed that cells on regions stiffer than the optimal stiffness would exert negative durotaixs [13,14,15]. The pathological relevance of negative durotaxis in tumor progression still requires in-depth investigation.

Recent work reported a distinct amplicon of YAP on the long arm of chromosome 11 in acral melanoma [16]. YAP is a mechanical-sensitive transcriptional coactivator [17], which can be activated by stiffer substrates and stronger contraction force [18,19]. YAP activation leads to increased cell proliferation, cell survival, and tumor transformation of mammary epithelial cells [20]. Meanwhile, YAP over-activation has been associated with tumor metastasis in multiple cancers [21,22]. Both single nucleotide mutation and gene structure variation have been reported in melanocytes to facilitate melanoma transformation [23], which typically occurs cutaneously, but may also develop in mucous membranes (mucosal melanoma) and eyes (uveal melanoma). Unlike ultraviolet induced cutaneous melanoma in Caucasians [24,25], the most common melanoma subtype in non-Caucasian patients is acral melanoma, which usually occurs on palms, soles and under the nails [26]. A clinicopathological analysis of acral melanoma reported that this particular type of melanoma tended to develop in stress-bearing areas [27]. However, how microenvironmental mechanics contributes to acral melanoma development and progression remains elusive.

In this study, we observed that YAP promotes the negative durotaxis of B16 F1. Mechanistic investigation further revealed that the RhoA-myosin II pathway may mediate YAP enhanced melanoma negative durotaxis. We further detected a decreasing stiffness gradient from the tumor to the surrounding area in the acral melanoma microenvironment. We demonstrated that the stiffness gradient could facilitate directed melanoma cell migration towards the soft region. Taken together, our work proposed negative durotaxis as a new mechanism for acral melanoma dissemination.

## 2. Methods and Materials

### 2.1. Acral Melanoma Samples

A total of 21 cases were collected from the Department of Pathology, Peking University Third hospital. All specimens were fixed in formalin and embedded in paraffin. This study was approved by the Research Ethics Committee (IRB00006761-M2021427), Peking University Third Hospital, Beijing, China.

### 2.2. Antibodies and Reagents

Anti-YAP (A1002) was purchased from ABclonal (Wuhan, China). Anti-mouse (sc-2005), and anti-rabbit (sc-2004) HRP conjugated secondary antibodies were purchased from Santa-Cruz Biotechnology (Dallas, TX, USA); anti-GAPDH (ab181602) was purchased from Abcam (Shanghai, China). The anti-pMLC (3674 s) was purchased from Cell Signaling Technologies (Denvers, CO, USA); anti-RhoA (ARH04) was purchased from Cytosketon (Denvers, CO, USA); anti-ARHGAP29 (sc-377022) was purchased from Santa-Cruz Biotechnology (Dallas, TX, USA); Alexa Fluor 488- or Alexa Fluor 555-conjugated secondary antibodies were obtained from Life (Carlsbad, CA, USA). Rho activator II were from Cytoskeleton. Myosin II inhibitor (blebbistatin) was from EMD_Millipore. Bromophenol Blue was generously donated by Prof. Yuxin Yin’s lab. Dimethyl sulfoxide (DMSO) was purchased from VWR (branch company in Shanghai, China). DNA transfection reagent was purchased from NEOFECT (Beijing, China).

### 2.3. Cell Culture

B16 F1 and F10 cells were purchased from the Cell Resource Center (IBMS, CAMS/PUMC, Beijing, China). HEK293T cells were kept by our laboratory. HEK293T cells were cultured in Dulbecco’s Modified Eagle Medium (DMEM) media supplemented with 10% fetal bovine serum (FBS), 100 U/mL penicillin and 100 µg/mL streptomycin. B16 F1 and B16 F10 cells were cultured in Roswell Park Memorial Institute (RPMI) 1640 media supplemented with 10% FBS, 100 U/mL penicillin and 100 µg/mL streptomycin. Cells were grown in a humidified incubator at 37 °C, under a 5% CO_2_ atmosphere and routinely checked for mycoplasma contamination. For cell passage, cells were washed once with PBS (Macgene, CC010, Beijing, China) and digested with 0.25% Trypsin-EDTA (Macgene, CC012, Beijing, China).

### 2.4. Plasmids and Stable Cell Line Generation

The pLKO.1 was obtained from Addgene. The knockdown efficiency was verified by western blot or qPCR. Three plasmid-packing system was used for lentivirus packing. Those three plasmids are pLKO.1 inserted with target genes, ps-PAX2 and pCMV-VSV-G. HEK293T cells were transfected following the Neofect DNA transfection protocol (KS2000). After 48 h, lentivirus can be harvest. Fresh lentivirus-containing media or enriched lentivirus were used to infect cell lines 3 times. Positive cells were selected by puromycin (Sigma-Aldrich, P8833, Saint Louis, MO, USA) at 1 μg/mL for 1 week, then keep the cell at 0.5 μg/mL.

Targeted sequences of knockdown are listed below.
Mouse YAP 1#TGAGAACAATGACAACCAATAMouse YAP 2#GAAGCGCTGAGTTCCGAAATCMouse ARHGAP29 1#GGATGCACTTAGTAGACATTTMouse ARHGAP29 2#CCAATTCCCTCGGAGCATTTAMouse NMHC IIA 1#CGGTAAATTCATTCGTATCAAMouse NMHC IIA 2#GCCATACAACAAATACCGCTTMouse NMHC IIB 1#CCTCCACAAGACATGCGTATTMouse NMHC IIB 2#CCGCTACTATTCAGGACTTATMouse MRLC2 1#GAGTATCTGGAGGGCATGATGMouse MRLC2 2#AGTTCACTCGCATCCTCAAAC

### 2.5. Fluorescence In Situ Hybridization (FISH) and Signal Measurement

FISH analysis was conducted as previously described using the Abnova YAP1/CEP11p FISH Probe Kit purchased from Thermo Fisher Scientific (PMID 29037804, Waltham, MA, USA). Samples were incubated with Alkaline Phosphatase AffiniPure Goat NegativeRabbit IgG (H + L) (Gene Technology, Shanghai, China) for 30 min. Centrosomes were marked by PermaRed/AP color-developing agent (Gene Technology, Shanghai, China) for 2 min of incubation. Nuclei were marked by DAPI. After hybridization, FISH slides were screened at high magnification (100 objective with oil immersion) for nuclei harboring abnormal copy numbers of either probe. A total of 30 non-overlapping intact tumor nuclei were counted for each slide. The percentage of cells with altered copy number and the average copy number for each gene site was calculated.

### 2.6. Immunohistochemistry and Evaluation of Immunostaining

YAP1 immunohistochemistry was performed with a LEICA BOND-MAX system using YAP1 (D8H1X) XP Rabbit monoclonal antibody (Cell Signalling Technology, Denvers, CO, USA). Evaluation of IHC staining took both the intensity of staining and the percentage of positive cells into account. Both plasma and nuclear staining was considered positive. 

### 2.7. Western Blot

For Western blotting, cells were washed with Dulbecco’s phosphate buffered saline (DPBS) once and lysed in an appropriate volume of radio immunoprecipitation assay (RIPA) buffer (50 mM Tris–HCl, pH 8.0, 150 mM NaCl, 1% Triton X-100, 0.5% Na-deoxycholate, 0.1% SDS, 1 mM EDTA and protease inhibitor cocktail (Thermo Fisher Scientific, 88666, Waltham, MA, USA) for 15 min on ice. Lysates were centrifuged at 12,000× *g* rpm for 10 min, and the supernatants were collected. 5 × SDS loading buffer was added to the supernatants and boiled for 10 min at 95 °C. To obtain GTP-RhoA, Rhotekin-RBD Protein GST Beads (Cytoskeleton, TR02) were used according to the manufacturer’s instructions. Protein samples were run on 10–12% SDS–PAGE acrylamide gels and transferred onto NC membranes by wet electrophoretic transfer, followed by first antibody incubation at 4 °C overnight or at room temperature for 2 h. Then, incubate with second antibody at room temperature for 1 h. The X-ray film was used to detect and record the band intensities. The fixed X-ray film was scanned, and digital images were obtained. The band intensity was quantified by “gel analysis” plugin of ImageJ.

### 2.8. Quantitative Real-Time PCR

Total RNA from patients’ acral lentiginous malenoma samples were isolated using Trizol (Life Technologies, 15596026, Carlsbad, CA, USA). RNA was extracted following protocol kept in our laboratory. RNA was reverse transcribed using a Transcript One-Step gDNA Removal and cDNA Synthesis SuperMix Kit (Transgene, AT311-02). Level of YAP, ANKRD1, CTGF and CYR61 genes were analyzed by quantitative Real-Time PCR (qRT-PCR) amplified using SYBR Green (ABclonal, RK21203, Wuhan, China). Data shown are the relative abundance of mRNA from patients’ melanoma samples normalized to mRNA from normal tissue samples.

Primers used in qRT-PCR are all in the list below.
Human CTGF FAGGAGTGGGTGTGTGGACGAHuman CTGF RCCAGGCAGTTGGCTCTAATCHuman YAP1 FTGCGTAGCCAGTTACCAHuman YAP1 RGGTGCCACTGTTAAGGAHuman ANKRD1 FAGTAGAGGAACTGGTCACTGGHuman ANKRD1 RTGGGCTAGAAGTGTCTTCAGATHuman CYR61 FAAGAAACCCGGATTTGTGAGHuman CYR61 RGCTGCATTTCTTGCCCTTTHuman GAPDH FAGGGCTGCTTTTAACTCTGGTHuman GAPDH RCCCCACTTGATTTTGGAGGGAHuman Actin FGATCATTGCTCCTCCTGAGCHuman Actin RACTCCTGCTTGCTGATCCAC

### 2.9. Immunofluorescence and Imaging Analysis

Cells were plated on PA gel coated with 10 μg/mL fibronectin overnight. Cells were then fixed with 4% paraformaldehyde (PFA) at room temperature for 15 min, permeabilized in 0.5% Triton X-100 in PBS for 10 min, washed with PBS three times for 5 min each time and blocked with 10% bovine serum albumin (BSA) for 1 h. Then, the primary antibody was diluted 1:200 or 1:100 in PBS and incubated for 1 h at room temperature. After washing with PBS three times and 5 min for each time, the coverslips were incubated with Alexa Fluor 488 or Flour 555 conjugated secondary antibody for 1 h at room temperature. Secondary antibodies were diluted in 1:200. The coverslips were then incubated with 0.2% phalloidin solution for 1 h. After another wash with PBS for three times, 5 min for each time, the coverslips were mounted with ProLong™ Glass Antifade Mountant with NucBlue™ Stain (P36981, Invitrogen, Carlsbad, CA, USA). After mounting medium was solidified, images were captured by Andor Dragonfly confocal imaging system.

The acral melanoma samples were sectioned into 30 μm-thick slices and attached onto glass slides. Slices were washed by DPBS containing 5% FBS and 0.2% Triton X-100 for 1 h. The primary antibodies of YAP were diluted in 1:100 with DPBS mix. The slides were incubated with the primary antibodies mix solution for 2 h at room temperature. After three times of DPBS washing, 5 min for each time, the slides were incubated with the secondary antibodies mix which was a solution of Fluor 488- and Fluor 555-conjugated secondary antibodies diluted in 1:100 with DPBS mix. The secondary antibodies mix solution also contained 0.4% phalloidin. After being incubated for 2 h, the slides were washed by DPBS three times, for 5 min each time. A coverslip was mounted onto the sample with ProLong™ Glass Antifade Mountant with NucBlue™ Stain (P36981, Invitrogen, Carlsbad, CA, USA). After mounting medium was solidified, images were captured by Andor Dragonfly confocal imaging system.

### 2.10. Atomic Force Microscope (AFM)

Acral melanoma stiffness measurement was performed on frozen samples. The samples were prepared as described [28]. The thickness of every sample was 30 μm. The sample was placed on AFM-compatible dishes. A Bioscope Resolve atomic force microscope (AFM; NT-MDT) was used to investigate the mechanical properties of acral melanoma. Silicon nitride probes with a squared pyramid tip (DNP, nominal cantilever spring constant = 0.06 N/m, Bruker, Billerica, MA, USA) were used in this study. 

### 2.11. Gradient Gel Generation and Functionalize

Polyacrylamide gels with a stiffness gradient were generated as described [29] with mild modifications. 65 μL acrylamide mix (19 μL 40% acrylamide, 19 μL 2% bis-acrylamide, 27 μL 10 mM HEPES with 2 mg/mL Irgacure2959, Sigma-Aldrich, 410896, Saint Louis, MO, USA) was applied to glutaraldehyde-modified 24 mm glass coverslip, covered with a glass coverslip made hydrophobic by treatment with Repel-Silane. Gradients were generated by initially covering the acrylamide mix solution with an opaque mask and then slowly sliding it at a controlled speed while irradiating with a UV bench lamp. The mask was slid with the help of an automatic syringe pump (Chemyx Fusion 200). To ensure complete polymerization, the whole acrylamide mix solution was first exposed to UV light for 12 min without covering, and then mask was slid at 40 μm/s for 10 min to produce the steep stiffness gradient gels. After gel photo-polymerization, the hydrophobic glass coverslip was removed and the gel was washed with PBS thoroughly to remove unreacted reagents. The stiffness was measured with AFM. To promote cell adhesion, fibronectin was covalently linked to the gels as described below. Uniform gels were made from 40% acrylamide and 2% bis-acrylamide mixed with 10% ammonium persulfate and 1% TEMED and received 20 min UV light explosion without any covering.

### 2.12. Spheroid Generation

B16 cells were counted, then centrifuged and resuspended in a concentration of 5000 cells per 100μL culture media. A total of 5000 cells (100μL resuspended solution) were added per well in a 96-well Corning Ultra-Low Attachment Spheroid Microplate (Corning) then incubated for 48 h. 

2 mg/mL collagen was coated on the bottom of a 12-well plate in a volume of 400 μL per well. The plate was then put into the incubator for 5 min. The supernatant of spheroid prepared before was abandoned. 30 μL of the remnant resolution of spheroid was then mixed with collagen gel uniformly and the 12-well plate was then put back to the incubator for 30 min. 1 mL culture media was added to each well of the plate. The spheroid would be used in the following imagining process after 1 h of incubating.

### 2.13. Collagen Gel Contractility Assay

Collagen was added into gel mix (10 × DPBS, 0.23% 1 N NaOH, and H_2_O) to generate the 2% collagen gel. B16 F1 cells were collected in a tube and centrifuged at 800× *g* rpm for 3 min. The cells were resuspended with 2% collagen gel at the density of 1×10^6^/mL. The cell mix was seeded into a 48-well plate and incubated in a cell incubator for 30 min. Appropriate volume medium was added into the wells. Photos of the collagen gels were taken at 0 h and 24 h. Fiji was used to count the area of collagen gels at each time point. Time-area curve was plotted by GraphPad Prism.

## 3. Results

### 3.1. YAP Promotes Negative Durotaxis

Directed cell migration play important roles in immune surveillance, embryo development and cancer metastasis [30,31]. The ability of cells to sense and migrate along gradients of substrate stiffness has been coined as durotaxis [10]. It has been reported recently that some cells managed to exert negative durotaxis or adurotactic migration [13,14].

**Figure 1:**
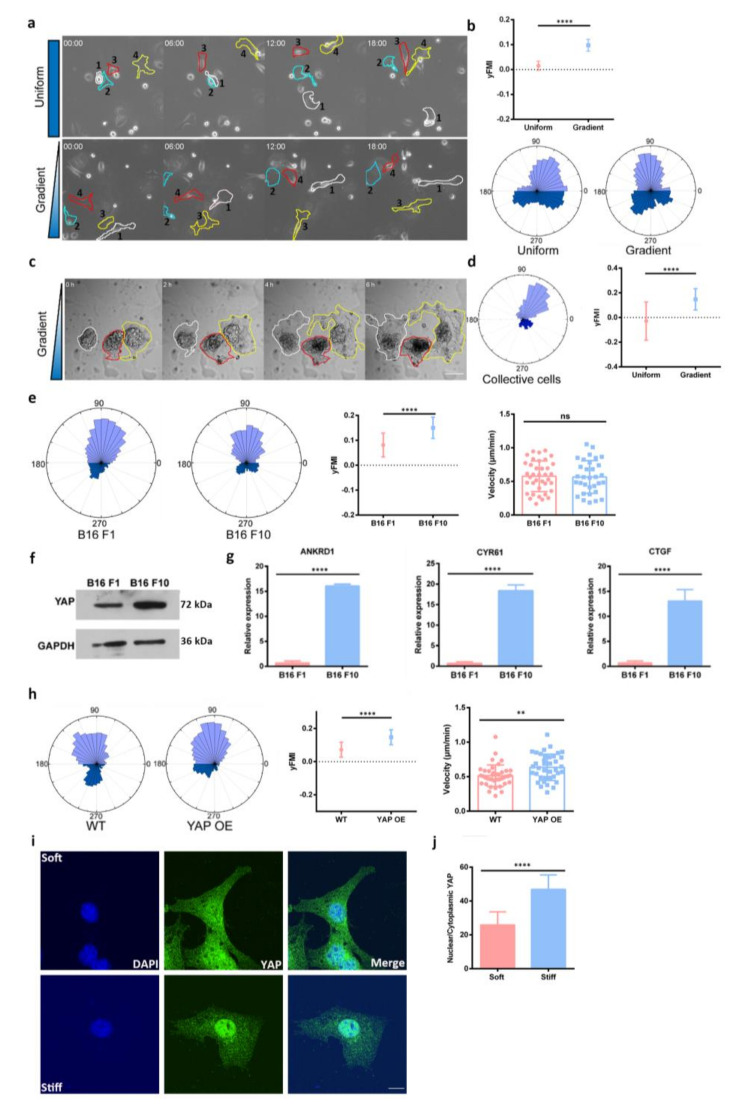
**YAP promotes negative durotaxis of melanoma cells.** (**a**) Representative time-lapse images showing the migration of single B16 F1 cells on the surface of polyacrylamide gels with uniform stiffness (top) and stiffness gradient (bottom), referring as random migration and durotaxis migration, Scale bar: 10 μm. (**b**) Upper right: Y-axis forward migration index (yFMI) of B16 F1 cell migration on stiffness gradient polyacrylamide (PA) gels, error bar is SD, ****, *p* < 0.0001, by student’s t test. Lower right: Rose plot on the left shows B16 F1 migration on uniform stiffness PA gel, rose plot on the right shows B16 F1 migration on stiffness gradient PA gel n_Uniform_ = 68, n_Gradient_ = 64. (**c**) Representative time-lapse images of collective B16 F1 cells migration on the surface of gradient PA gel, the three spheroids were outlined by lines in different colors, Scale bar: 100 μm. (**d**) Left: Rose plot of collective B16 F1 cell migration; Right: yFMI of collective B16 F1 cell migration on different stiffness PA gels, n = 24, ****, *p* < 0.0001, by student’s t test. (**e**) Left: Rose plot of B16 F1 and F10 cell migration on stiffness gradient PA gel; Right: yFMI of B16 F1 and F10 cell migration on stiffness gradient PA gel, n_B16 F1_ = 37, n_B16 F10_ = 32, error bar is SD, ****, *p* < 0.0001, ns, no significant difference, by student’s t test. (**f**) Western blot shows YAP protein level of B16 F1 and F10 cells, GAPDH is used as loading control. (**g**) mRNA level of YAP target genes (CYR61/CTGF/ANKRD1) in B16 F1 and B16 F10 cell lines, error bar is SEM, ****, *p* < 0.0001, by student’s t test. (**h**) Left: Rose plots of B16 F1 wild type (WT)/YAP overexpression (YAP OE) migration on stiffness gradient PA gel. Right: yFMI, velocity of B16 F1 Wildtype (WT)/YAP overexpression (YAP OE) migration on stiffness gradient PA gel, n_WT_ = 40, n_YAP OE_ = 39, error bar is SD, ns, none significant difference, by student’s t test, ****, *p* < 0.0001, **, *p* < 0.01, by student’s t test. (**i**) Representative images of YAP localization in B16 F1 cells on the gradient gel, blue: nucleus, green: YAP, scale bar: 10 μm; (**j**) Quantification of nuclear/cytoplasmic YAP, n_Stiff_ = 9, n_Soft_ = 11, ****, *p* < 0.0001, by student’s t test.

By using an established protocol [12,29], we manufactured PA gel with the 15 kPa/mm stiffness gradient. The gradient PA gel was then functionalized with fibronectin or laminin before seeding the B16 F1 melanoma cells. Live cell imaging and the subsequent tracking of cell migration were performed to record cell migration activities. Interestingly, we found that B16 F1 cells tended to migrate from the stiff area to the soft area of the gel, which was contrary to durotaxis called negative durotaxis (Figure 1a,b and Figure S1a, Videos S1 and S2). To probe whether collective B16 F1 cells also exhibit negative durotaxis, we employed the spheroid migration assay. B16 F1 spheroids were generated following an established protocol before being plated onto the stiffness gradient gel for live cell imaging and cell migration analysis. Indeed, similar soft-side-directed migration was observed in these B16 F1 spheroids (Figure 1c,d and Figure S1b, Video S3). B16 F10 cells are obtained by a 10-time selective procedure using the Fider’s method and appear to be more invasive than B16 F1. Furthermore, we asked whether the invasion capacity may coincide with the extent of negative durotaxis by comparing the forward migration index (FMI) of B16 F1 cells with that of the B16 F10 cells. Our analysis suggested that B16 F10 exhibited a stronger tendency to undergo negative durotaxis (Figure 1e and Figure S1c). Incidentally, we found that YAP abundance is higher in B16 F10 cells compared to B16 F1 (Figure 1f). Increased mRNA level of YAP target genes in B16 F10 cells was also detected by qRT-PCR (Figure 1g). To address whether the difference in negative durotaxis migration between B16 F1 and B16 F10 cells is related to the differed YAP expression, we overexpressed YAP in B16 F1 cells and monitored cell migration on stiffness gradient PA gel. Interestingly, this manipulation increased the forward migration index (FMI) of B16 F1 cells to the level comparable with B16 F10 (Figure 1h and Figure S1d), indicating enhanced negative durotaxis. To gain insight of YAP in negative durotaxis, we investigated the subcellular localization of YAP in B16 F1 cells on gradient gel. We found that YAP translate from nucleus to cytoplasm as the stiffness decreases (Figure 1i,j). Together, these results suggested that YAP may promote negative durotaxis of melanoma cells.

### 3.2. RhoA and Myosin II Mediate YAP-Promoted Negative Durotaxis 

**Figure 2:**
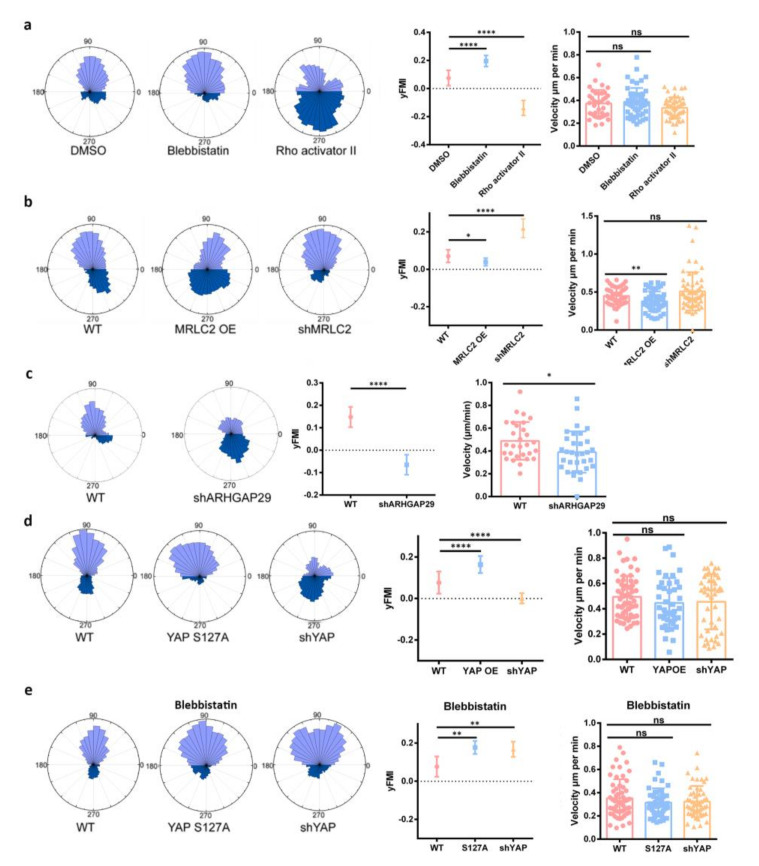
**YAP promotes negative durotaxis through ARHGAP29-RhoA-Myosin II.** (**a**) Left: Rose plots of B16 F1 cell migration on stiffness gradient PA gel after treated with DMSO/blebbistatin/Rho activator II. Right: yFMI, velocity of B16 F1 cell migration on stiffness gradient PA gel after treated with DMSO/blebbistatin/Rho activator II, n_DMSO_ = 38, n_blebbistatin_ = 53, n_Rho activator II_ = 51, error bar is SD, ****, *p* < 0.0001, by one-way ANOVA, ns, none significant, by student’s t test. (**b**) Left: Rose plots of B16 F1 WT/MRLC2 OE/shMRLC2 cell migration on stiffness gradient PA gel. Right: yFMI, velocity of B16 F1 WT/MRLC2 OE/shMRLC2 cell migration on stiffness gradient PA gel,n_WT_ = 46, n_MRLC2_ = 53, n_shMRLC2_ = 59, error bar is SD, ****, *p* < 0.0001, **, *p* < 0.01, *, *p* < 0.05,ns, no significant difference, by one-way ANOVA, ns, none significant, by student’s t test. (**c**) Left: Rose plots of B16 F1 WT/shARHGAP29 cell migration on stiffness gradient PA gel. Right: yFMI, velocity of B16 F1 WT/shARHGAP29 cell migration on stiffness gradient PA gel, n_WT_ = 29, n_shARHGAP29_ = 32 error bar is SD, by student’s t test. (**d**) Left: Rose of B16 F1 WT/YAP S127A/ shYAP cell migration on stiffness gradient PA gel; Right: yFMI, velocity of B16 F1 WT/YAP S127A/shYAP migration on stiffness gradient PA gel, n_WT_ = 57, n_S127A_ = 42, n_shYAP_ = 45, error bar is SD, ****, *p* < 0.0001, *, *p* < 0.05, by one-way ANOVA, ns, none significant, by student’s t test. (**e**) Left: Rose of B16 F1 WT/YAP S127A/shYAP cell migration on stiffness gradient PA gel after treated with blebbistatin; n_WT_ = 56, n_S127A_ = 48, n_shYAP_ = 51, Right: yFMI, velocity of B16 F1 WT/YAP S127A/shYAP cell migration on stiffness gradient PA gel after treated with blebbistatin, error bar is SD, **, *p* < 0.01, ns, no significant difference, by one-way ANOVA, ns, none significant, by student’s t test.

Next, we set to explore the mechanism underlying YAP-enhanced melanoma negative durotaxis. YAP has been reported to influence actin dynamics by increasing ARHGAP29 expression and thus decreasing RhoA activity [32]. We speculated that one possible mechanism for YAP to regulate negative durotaxis may be through its negative regulation of RhoA. To probe this, we treated cells with Rho activator II and examined cell migration on stiffness gradient gel. Decreased FMI indicated that RhoA activation hampered negative durotaxis (Figure 2a and Figure S2a). RhoA can activate myosin II through ROCK signaling pathway. In agreement with previous finding [14], we observed increased FMI when we inhibited the activity of myosin II by blebbistatin in B16 F1 cells (Figure 2a and Figure S2a). Moreover, myosin II activation through MRLC2 overexpression inhibited negative durotaxis while myosin II inhibition through MRLC2 knock-down promoted negative durotaxis (Figure 2b and Figure S2b).

Consistent with previous findings [32], we observed that YAP overexpression inhibited RhoA activity and increased the expression of ARHGAP29 by Western blot (Figure S2c). YAP overexpression also decreased the ability of melanoma cells to contract collagen gels (Figure S2d). Moreover, ARHGAP29 knock-down inhibited negative durotaxis (Figure 2c and Figure S2e,f). In order to test whether YAP promotes negative durotaxis through ARHGAP29-RhoA-myosin II pathway, we inhibited myosin II activity and detected whether disturbing YAP activity could still influence negative durotaxis. When YAP was knocked down, the tendency of negative durotaxis was decreased as cells were more likely to migrate adurotactically when YAP S127A was expressed in B16 F1 cells (Figure 2d and Figure S2g,h). Interestingly, the inhibition of myosin II could barely affect negative durotaxis regardless of intracellular YAP activity (Figure 2e and Figure S2i). Together, these observations indicate that RhoA-myosin II pathway is critical for YAP enhanced soft side biased migration in melanoma cells.

### 3.3. Acral Melanoma Exhibit Increased YAP Activity

**Figure 3:**
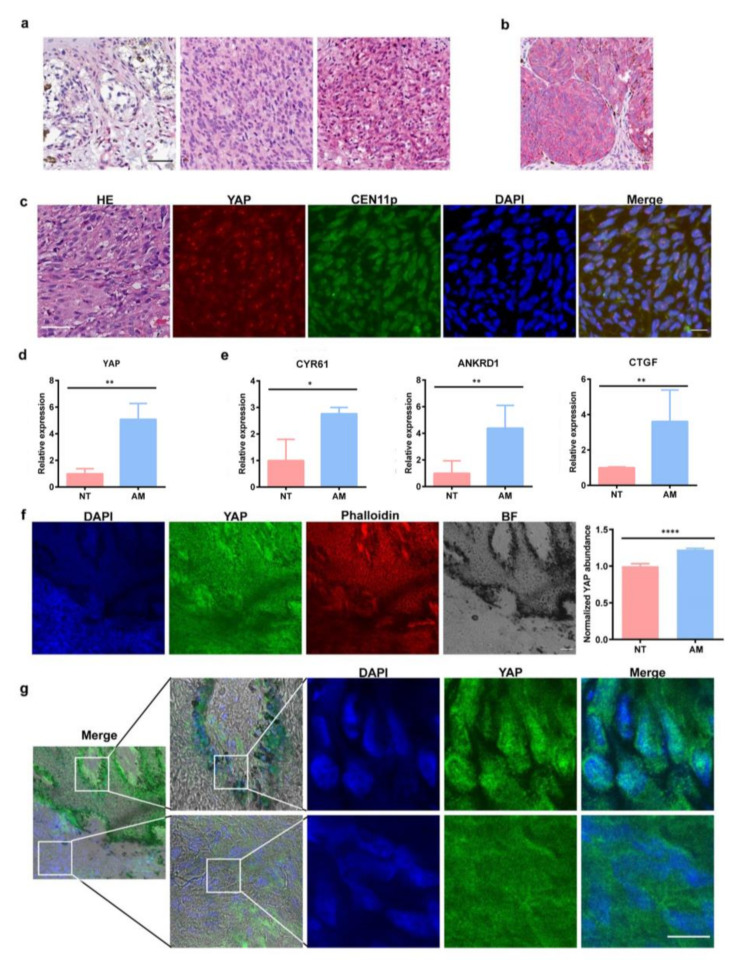
**Acral melanoma patients exhibit YAP amplification and increased YAP activity.** (**a**) Immunohistochemistry images from different samples showing different expression pattern of YAP protein. Left: YAP is negative in both cytosol and nucleus. Middle: YAP is positive in cytosol but negative in nucleus. Right: YAP is positive in both cytosol and nucleus, Scale bar: 50 μm. (**b**) Immunohistochemistry image of the only one YAP amplification sample of acral melanoma patient, Scale bar: 50 μm. (**c**) Immunohistochemistry showing YAP protein level and FISH showing YAP amplification level from 2 in 21 samples of acral melanoma patients. Immunohistochemical result was another different sample from that of FISH results. YAP (Red), centrosomes (Green) and nuclei (Blue). Scale bar: 50 μm. (**d**) mRNA level of YAP in normal tissue (NT) and acral melanoma (AM), error bar is SEM, n(NT) = 4, n(AM) = 4, *, *p* < 0.05, **, *p* < 0.01. (**e**) mRNA level of YAP target genes (CYR61/CTGF/ANKRD1) in normal tissue and acral melanoma, error bar is SEM, n(NT) = 4, n(AM) = 4. (**f**) Left: Representative images of YAP in normal tissue and acral melanoma, nucleus (Blue), YAP (Green), actin (Red) and bright field (BF). Scale bar: 100 μm. Right: Quantification of YAP intensity in normal tissue and acral melanoma, error bar is SEM, ****, *p* < 0.0001, by student’s t test. (**g**) Zoomed in images of YAP in normal tissue and acral melanoma, nucleus (Blue), YAP (Green). Scale bar: 10 μm. The upper lane of images is zoomed from melanoma region, while the lane of images below is from normal tissue region of the same sample.

To investigate the pathological relevance of YAP and negative durotaxis in tumor progression, we searched the Cancer Genome Atlas (TCGA) database and found that melanoma patients with higher YAP expression exhibit poor survival (Figure S3a). We collected acral melanoma samples from 21 patients under permission. All these samples were evaluated for YAP1 protein expression using immunohistochemistry (IHC), the specificity of YAP was tested (Figure S3d). Twelve of these patients were male and nine were female. The median patient age was 62 years with a range of 33 to 87 years (Table S1). The mean Breslow thickness was 5.3 mm (range 0.9 mm to 30.0 mm, Table S2). Ulceration was observed in seven cases. Two cases of acral melanoma in situ showed totally YAP1 negative. The other 19 acral melanomas, including two melanomas in situ and 17 invasive melanomas, showed YAP1 expression. Nine cases showed only cytoplasm expression (Table S2). Ten cases showed cytoplasm expression combined with focal nuclear expression (Figure 3a). No cases showed exclusive nuclear expression.

We collected 10 samples randomly from those samples used in the previous IHC evaluations. These samples were then evaluated for YAP1 gene amplification using fluorescence in situ hybridization (FISH), in which four patients were male and six were female. The median patient age was 71 years (ranging from 57 to 87 years, Table S1). The mean Breslow thickness was 2.3 mm (ranging from 0.9 mm to 4.0 mm, Table S1). Ulceration was observed in four cases. A recent study has reported that some acral melanoma patients (12.3%) harbor YAP amplification [23]. In our samples, one case (1/10, 10%) exhibited YAP1 amplification (Figure 3b). The patient was a 57 years old female, and the tumor was located on the left toe. Nine cases showed normal copy number of YAP1 (Figure S3b). The only one YAP1 amplification acral melanoma case in our cohort showed diffuse plasma expression of YAP1 protein (Figure 3c). Nine cases with normal YAP1 copy numbers showed non YAP1 expression in 1 case (1/9), only cytoplasm expression in four cases (4/9) and cytoplasm expression combined with focal nuclear expression in four cases (4/9). These observations in patients and fixed samples support the notion that YAP may not only be amplified but is over activated in acral melanoma.

We also evaluated YAP expression level from collected pairwise samples of acral melanoma and surrounding normal tissues. First, we detected that YAP mRNA abundance increased in melanoma samples (Figure 3d). Moreover, the mRNA levels of three standard downstream effectors of YAP-CYR61, CTGF and ANKRD1 also elevated (Figure 3e), indicating that YAP activity may be enhanced in these samples. Second, we found that YAP protein level was much higher in acral melanoma region than in normal area by immunofluorescence (Figure 3f). We also noticed that YAP exhibited obvious nuclear localization in acral melanoma samples, while it dispersed in the cytoplasm in normal tissues (Figure 3g and Figure S3c). 

### 3.4. Acral Melanoma Provides a Perfect Mechanical Environment for Tumor Invasion through Negative Durotaxis

**Figure 4:**
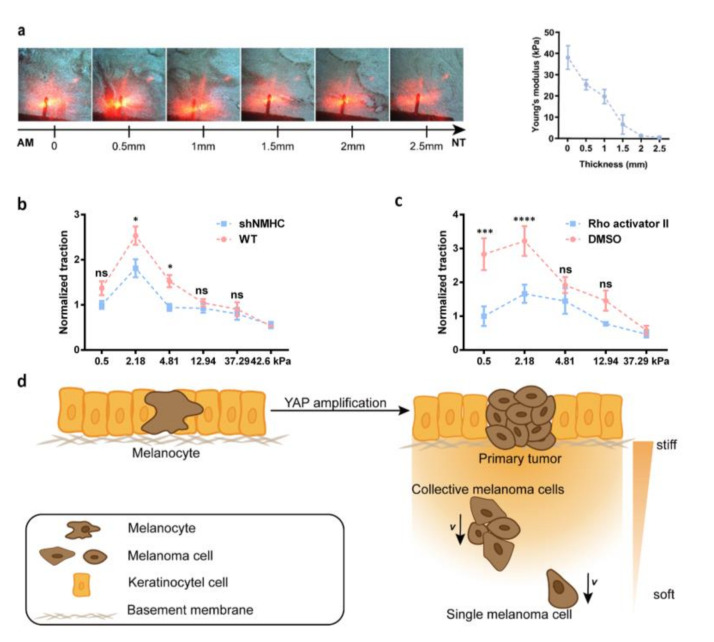
**Acral melanoma provides a perfect mechanical environment for tumor invasion though negative durotaxis.** (**a**) Left: Images shows stiffness measurement using atomic force microscopy (AFM), red/yellow color is laser of AFM, the shadow in the center of red/yellow color is the cantilever of AFM, AM: acral melanoma, NT: normal tissue; Right: Quantification of acral melanoma stiffness every 0.5mm, error bar is SD, *, *p* < 0.05, by one-way ANOVA, ns, none significant. (**b**) Tracktion force microscopy (TFM) test traction force of B16 F1 WT/shNMHC on different stiffness gel, error bar is SD, *, *p* < 0.05, ns, no significant difference, by one-way ANOVA. (**c**) TFM test traction force of B16 F1 treated with DMSO/Rho activator II on different stiffness gel, error bar is SD, ***, *p* < 0.001, ****, *p* < 0.0001, ns, no significant difference. (**d**) Schematic diagram of YAP activation in promoting anti-durotaxis and acral melanoma progression.

To explore the role of biomechanical environment in acral melanoma invasion, we measured the stiffness of acral melanoma at the invasive border in a more precise and continues way and a stiffness gradient of 15 kPa/mm was found along the tumor -normal tissue axis using AFM (Figure 4a). These observations in acral melanoma provided a perfect mechanical environment for acral melanoma invasion though negative durotaxis.

It has been documented that the motor-clutch model explains the negative durotaxis of U251MG cells. We thus also tested if the negative durotaxis of B16 F1/F10 cells can be explained by similar motor-clutch model. By using traction force microscopy, we found that B16 F1 has maximum traction force on the stiffness of 2.18 kPa (Figure 4b), indicating that B16 F1 exhibits maximal traction at an optimal stiffness of 2.18 kPa. The knockdown of NMHC or activation of myosin II by Rho activator II did not disturb the “optimal stiffness” of B16 F1 (Figure 4b,c and Figure S3e), suggesting that disturbing motor number did not switch B16 F1/F10 from negative durotaxis to positive durotaxis in our stiffness gradient gel system.

## 4. Discussion

Our study suggested that YAP activation may play a critical role in acral melanoma progression. We unveiled the anti-durotactic behavior of melanoma cells and proposed that negative durotaxis may involve in melanoma invasion (Figure 4d). We further interrogated the underlying mechanism and identified the contribution of YAP-RhoA-myosin II pathway in melanoma negative durotaxis regulation; and for the lack of acral melanoma cell line, whether acral melanoma invasion through negative durotaxis should be further investigated. Our work may shed new lights on the development of new therapeutic strategies for acral melanoma from a biomechanical perspective.

Skin homeostasis relies on the balance between intrinsic and extrinsic mechanical force, represented by cytoskeleton, ECM, intracellular signaling and the external stress applied [33]. The mechanical dysfunction of skin impacts fundamental biological process such as cell differentiation and proliferation. We observed stiffness gradients of acral melanoma, which decreases from the internal part to the peritumor area (Figure 4a). Whether this stiffness gradient has impacts on the reorganization of extracellular matrix and the cell proliferation pattern is worth investigating in the future.

Having observed that mechanical force may contribute to acral melanoma progress, it is intriguing to ask which mechanosensitive proteins contributes to acral melanoma progression. Previous work reported that acral melanoma tended to have more gene structure variation [25]. Among the numerous gene structure variation, we found that YAP was amplificated and had a higher proliferation rate in the peritumor area than normal tissue. Moreover, YAP promotes the negative durotaxis of acral melanoma. According to the motor-clutch model, which is composed of F-actin and myosin II, blocking adhesion reinforcement shifts cells from positive to negative durotaxis [34]. However, YAP overexpression promotes focal adhesion formation through RhoA [35,36], which indicates that YAP overexpression may not block the adhesion reinforcement of cells. Thus, YAP overexpression may not shift cells from positive to negative durotaxis. Moreover, by using traction force microscopy, we found that B16 F1/F10 cells have maximum traction force on the stiffness of 2.18 kPa. The knock down of non-muscle myosin-II or elevated myosin II activity did not disturb the “optimal stiffness” of B16 F1. This indicates that disturbing motor number did not switch B16 F1/F10 from negative durotaxis to positive durotaxis in this stiffness range.

Piezo proteins have been reported to mediate mechano-transduction [37,38]. The activation of piezo channels triggers the intracellular Ca^+^ signaling pathway, which plays pivotal roles in tissue homeostasis [39,40]. It has been reported that the activation of the Piezo1/Ca^+^/PDE1/PKA pathway promotes the confined migration of invasive melanoma cells [41], the role of piezo1 in linking melanoma to the micro-environment and tumor progression remains largely unexplored [42]. Cadherin complexes are also mechano-transducers that sense changes in tension and trigger the adaptive reinforcement of intercellular junctions [43]. Cadherin responds to both endogenous and exogenous forces. The knockdown of N-cadherin inhibits the invasion of human melanoma cells [44]. Whether Cadherin complexes mediated mechano-sensation in acral melanoma is worth investigating in the future.

In this study, we have tested two different melanoma cell lines-B16 (a mouse melanoma cell line) and A375 (a human melanoma cell line) and found negative durotaxis in both cell lines. However, we failed to get an acral melanoma cell line. It will be of critical interest to test negative durotaxis in cell lines that represents specific subtypes of melanoma.

Here, we proposed an important role of the tumor mechano-environment in acral melanoma progression and dissemination. A more precise and thorough view of the tumor micro-environment and better modeling of tumor-stroma-ECM mechano-interaction may bring new knowledge to our understanding of the physical property and reactivity of tumors.

## Data Availability

Not applicable.

## Supplementary Materials

The following supporting information can be downloaded at: https://www.mdpi.com/article/10.3390/cells11223543/s1, Figure S1: B16 undergoes negative durotaxis; Figure S2: YAP overexpression increases the activity of ARHGAP29 and cell contractility; Figure S3: Acral melanoma patients exhibit YAP amplification and increased YAP activity; Table S1: YAP protein expression lever and expressing pattern diversities observed in patients; Table S2: YAP gene copy number diversities observed in patients.
