# Supplementary material for: YAP Activation in Promoting Negative Durotaxis and Acral Melanoma Progression
Source: Cells. 2022 Nov 9;11(22):3543. doi: 10.3390/cells11223543 (PMC9688430; doi:10.3390/cells11223543)
Supplement: Supplementary file 1 [file cells-11-03543-s001.zip › Supplemental materials 20221106.pdf]

Supplemental materials:

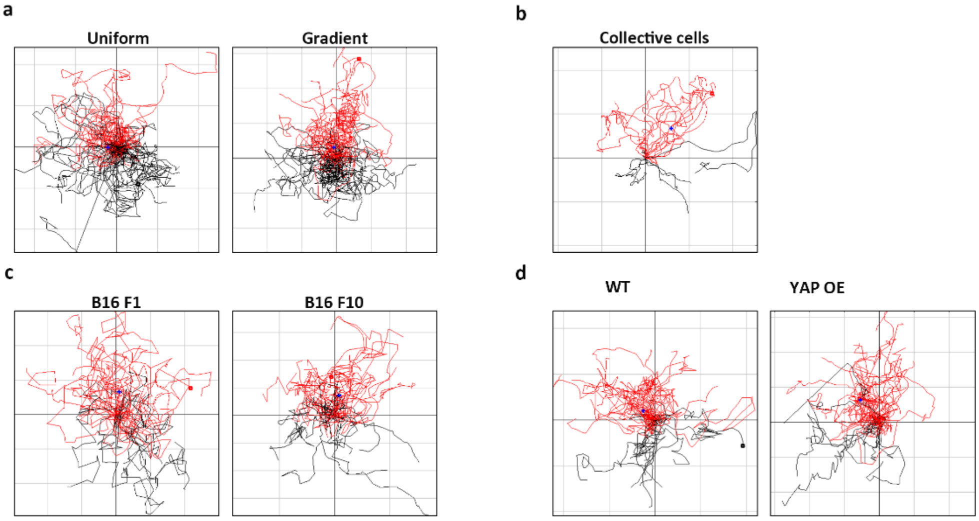

Figure S1 B16 undergoes negative durotaxis

a. Spider plot shows B16 F1 migration on uniform stiffness PA gel, $n_{Uniform}$=68, $n_{Gradient}$=64;
b. Spider plot of collective B16 F1 cell migration on stiffness gradient PA gels, n=24;
c. Spider plot of B16 F1/F10 cell migration on stiffness gradient PA gels, $n_{B16\ F1}$=37, $n_{B16\ F10}$=32;
d. Spider plot of B16 F1 WT and YAP overexpression cell migration on stiffness gradient PA gels, n_{WT}=40, $n_{YAP\ OE}$=39.

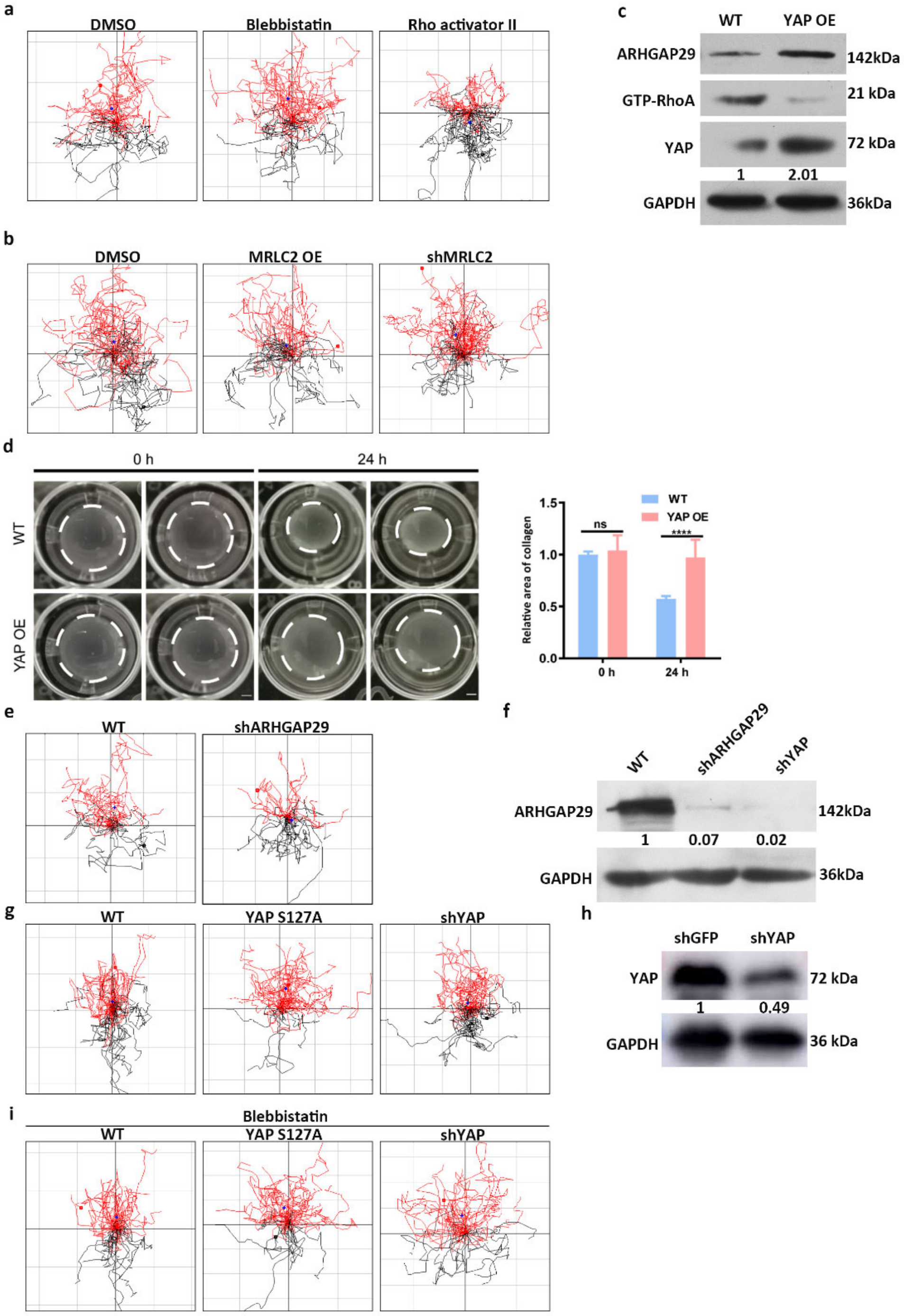

Figure S2 YAP overexpression increases the activity of ARHGAP29 and cell contractility

a. Spider plot of B16 F1 cell migration on stiffness gradient PA gels after DMSO/Blebbistatin/Rho activator II treatment, n_{DMSO}=38, $n_{blebbistatin}$=53, $n_{Rho\ activator\ II}$=51;

b. Spider plot of B16 F1 WT/MRLC2 OE/shMRLC2 migration on stiffness gradient PA gel, n_{WT}=46, n_{MRLC2}=53, $n_{shMRLC2}$=59;

c. Western blot showing the protein level of ARHGAP29, GTP-RhoA,YAP and total RhoA in WT and YAP-overexpression (YAP OE) B16 F1 cells. GAPDH was used for loading control. Middle and Right: The normalized abundance of ARHGAP29 and GTP-RhoA in WT and YAP OE B16 F1 cells;

d. Left: Images showing collagen deformation at 0, 24h. B16 F1 WT and YAP OE cells were seeded in 1% collagen, scale bar: 1mm. Right: Quantification of collagen area, error bar is SD, ns, no significant difference, ****, p<0.01, by student's t test;
e. Spider plot of B16 F1 WT/shARHGAP29 cells migration on stiffness gradient PA gel, n_{WT}=29, $n_{shARHGAP29}$=32;
f. Western blot showing the protein level of ARHGAP29 in negative control, YAP and ARHGAP29 knockdown B16 F1 cells;
g. Spider plot of B16 F1 WT/YAP S127A/ shYAP migration on stiffness gradient PA gel, n_{WT}=57, n_{S127A}=42, n_{shYAP}=45;
h. Western blot showing the protein level of YAP in negative control and YAP knockdown B16 F1 cells;
i. Spider plot of B16 F1 WT/YAP S127A/ shYAP migration on stiffness gradient PA gel after blebbistatin treatment, n_{WT}=56, n_{S127A}=48, n_{shYAP}=51.

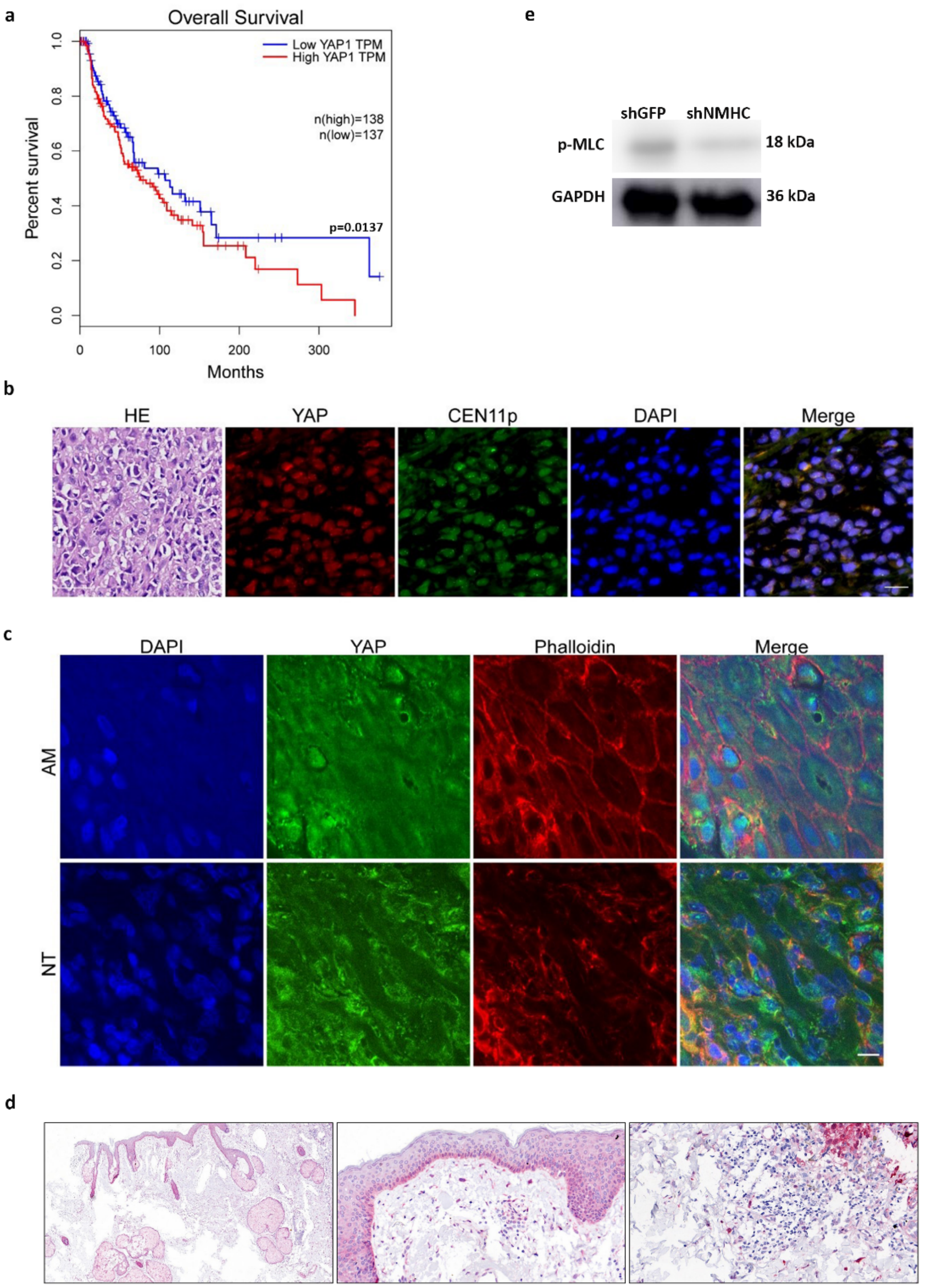

Figure S3 Acral melanoma patients exhibit YAP amplification and increased YAP activity

a. Overall survival of melanoma patients with different expression of YAP from TCGA data base, n (high expression) =138, n (low expression) =137;

b. Representative immunohistochemical images and FISH images from different samples of acral melanoma patients. YAP (Red), centrosomes (Green) and nuclei (Blue), Scale bars: 50 μm;

c. Representative images of YAP in normal tissue (NT) and acral melanoma (AM), nucleus (Blue), YAP (Green), actin (Red). Scale bar: 10 μm;

d. Left: IHC staining for YAP in squamous cells, sebaceous glands and hair follicle; Middle: IHC staining for YAP in normal melanocytes and dermal fibroblasts; Right: IHC staining for YAP in lymphocytes;

e. Western blot showing protein level of negative control and NMHC knockdown B16 F1 cell.

Table S1

	Cases number (Percentage)	Gender ratio (Male/Female)	Median age (year)	Ulceration cases (Percentage)	Mean Breslow Thickness (mm)
Only cytoplasm YAP expression	9 (43.9%)	-	-	-	-
Cytoplasm & nuclear YAP expression	10 (47.6%)	-	-	-	-
Negative YAP protein expression	2 (9.5%)	-	-	-	-
Total	21 (100%)	1.33	62	7 (33.3%)	5.3

Table S1. YAP protein expression lever and expressing pattern diversities observed in patients. This table shows the basic information of 21 patients agreeing to participate in our research, as well as their distribution in different intracellular YAP expressing patterns.

Table S2

	Cases number (Percentage)	Gender ratio (Male/Female)	Median age (Year)	Ulceration cases (Percentage)	YAP expression cases (Percentage)	Intracellular YAP expression pattern	Mean Breslow Thickness (mm)
YAP gene amplification	1 (10%)	0	57	-	1 (11.1%)	Only in Cytoplasm	-
Normal YAP copy number	9 (90%)	0.8	-	-	8 (88.9%)	50% only in cytoplasm, 50% in both cytoplasm and nuclear	-
Total	10 (100%)	0.67	71	4 (40%)	9 (90%)		2.3

Table S2. YAP gene copy number diversities observed in patients. This table shows the basic associated information of 10 patients agreeing to participate in our research.
